# Integrated care model for patients with functional somatic symptom disorder – a co-produced stakeholder exploration with recommendations for best practice

**DOI:** 10.1186/s12913-024-11130-9

**Published:** 2024-06-03

**Authors:** Frank Röhricht, Carole Green, Maria Filippidou, Simon Lowe, Nicki Power, Sara Rassool, Katherine Rothman, Meera Shah, Nina Papadopoulos

**Affiliations:** 1 East London NHS Foundation Trust (ELFT), London, United Kingdom; 2 Queen Mary University of London, London, United Kingdom; 3 Bedfordshire Community Health Services (BCHS), Bedford, United Kingdom; 4 Bedford Liaison Psychiatry Service, ELFT, Bedford, United Kingdom; 5 Circle Bedfordshire Integrated Care Musculoskeletal Service, Bedford, United Kingdom; 6 Clinical Health Psychology Services Bedfordshire & Luton, ELFT, Dunstable, United Kingdom; 7 Bedfordshire & Luton Community Adult Mental Health & Learning Disability Services , ELFT, Clapham, United Kingdom; 8 Clinical Health Psychology Service, Cambridge University Hospitals NHS Foundation Trust, Cambridge, United Kingdom

**Keywords:** Functional somatic symptoms and disorders, Medically unexplained symptoms, Psychosomatic medicine, Somatoform disorders, Somatic symptom disorder, Bodily distress disorder, Embodiment, Body-oriented psychological therapy

## Abstract

**Background:**

Functional somatic symptoms (FFS) and bodily distress disorders are highly prevalent across all medical settings. Services for these patients are dispersed across the health care system with minimal conceptual and operational integration, and patients do not currently access therapeutic offers in significant numbers due to a mismatch between their and professionals’ understanding of the nature of the symptoms. New service models are urgently needed to address patients’ needs and to align with advances in aetiological evidence and diagnostic classification systems to overcome the body–mind dichotomy.

**Method:**

A panel of clinical experts from different clinical services involved in providing aspects of health care for patients with functional symptoms reviewed the current care provision. This review and the results from a focus group exploration of patients with lived experience of functional symptoms were explored by the multidisciplinary expert group, and the conclusions are summarised as recommendations for best practice.

**Results:**

The mapping exercise and multidisciplinary expert consultation revealed five themes for service improvement and pathway development: time/access, communication, barrier-free care, choice and governance. Service users identified four meta-themes for best practice recommendations: focus on healthcare professional communication and listening skills as well as professional attributes and knowledge base to help patients being both believed and understood in order to accept their condition; systemic and care pathway issues such as stronger emphasis on primary care as the first point of contact for patients, resources to reduce the length of the patient journey from initial assessment to diagnosis and treatment.

**Conclusion:**

We propose a novel, integrated care pathway for patients with ‘functional somatic disorder’, which delivers care according to and working with patients’ explanatory beliefs. The therapeutic model should operate based upon an understanding of the embodied nature of patient’s complaints and provide flexible access points to the care pathway.

**Supplementary Information:**

The online version contains supplementary material available at 10.1186/s12913-024-11130-9.

## Background

Healthcare systems globally struggle to provide for patients who present with functional somatic symptoms. Burton et al. [1, p. 1], on behalf of the EURONET-Soma group, acknowledge that “functional somatic symptoms and disorders are common and complex phenomena…they pose major challenges across medical specialities”. Accordingly, the National Health Service (NHS) in the United Kingdom emphasises on its website that “many people have persistent physical complaints”. Although Burton et al. proposed “a new classification, ‘functional somatic disorder’, which is neither purely somatic nor purely mental” (p. 1), the frequently used term “Medically Unexplained Symptoms” (MUS) carries the assumption that they “…don’t appear to be symptoms of a medical condition…” [2]. This notion is out of date with respect to developments in the understanding of functional somatic symptom disorders; the new versions of the two main international diagnostic classification systems – DSM-V and ICD-11 [3, 4] – no longer differentiate between medically un/explained somatic disorders; instead, they refer to the ongoing presence of somatic symptoms that are distressing and result in excessive attention under the new terms somatic symptom disorder/bodily distress disorder (SSD/BDD). In fact, a large proportion of patients with long-term medical conditions suffer from bodily distress symptoms, and the criterion of ‘no physical basis’ has consequently dropped in line with new findings regarding the aetiology of the disorder, updating the biopsychosocial model [5, 6]. On that basis we decided to choose “functional somatic symptom disorder” as umbrella term whilst reporting findings from the literature according to the terms used in the primary literature. The incidence reported under the terms MUS or persistent physical symptoms (PPS) suggests that at least 20% of primary care presentations account for these conditions, approximately 50% in secondary medical outpatient clinics [7, 8] and that patients with MUS/PPS have disproportionately high rates of healthcare utilisation [9]. An estimated 10% of the annual expenditure working-age population in the NHS in England (at least three billion £ each year) is used to diagnose and treat MUS/PPS, resulting in appr. £1.2k per patient annually on average [9, 10]. The total societal costs are estimated to be approximately £18 billion [11]. The Kings Fund [12] analysed the situation across the NHS and concluded, “Much of this expenditure currently delivers limited value to patients; at worst, it can be counterproductive or even harmful” (p. 10). It has been estimated that 70% of people with MUS/PPS will also experience comorbid depression or anxiety disorders [2]; hence, there is a compelling case for delivering care in an integrated way to ensure that a person’s psychosocial and physical health and needs are met. Despite the enormous impact on service, pressures on health economies and poor treatment outcomes, there is currently no dedicated/specific integrated care pathway at the primary or secondary care level in the NHS for patients with functional somatic disorders. The standard talking therapy offered within NHS talking therapy services (formerly known as ‘Improving Access to Psychological Therapy’/IAPT services) for patients with MUS and Long-Term-conditions (LTC) delivers psychological (“talking”) therapy through a separate provider entity, and the treatment offered is not accepted by a large proportion of referred patients; drop-out rates are 50%, and a recent estimate of only 17% recovery rates [13, 14] indicates the need for a more efficient and acceptable treatment. Existing care models for patients with functional somatic syndromes do not adequately address the needs of these patients. New and innovative intervention strategies are necessary to achieve better health and corresponding economic outcomes. Therefore, experts have suggested that research into new, effective, and favourable service models and treatments that are acceptable to patients according to their preferences and specific explanatory beliefs is urgently needed [15].

The East London Foundation Trust (ELFT) provides mental, community and primary health services across the Luton and Bedfordshire geographical footprint (Bedford, Luton, Milton Keynes / BLMK Integrated Care System). Between 2010 and 2020, ELFT clinicians developed and evaluated a novel care pathway package [16–18] and a manualised body-oriented group therapy intervention [19] for patients with MUS disorders, which is now implemented in East London. The service demonstrated that this novel, embedded care package benefited patients and contributed to reductions in health care utilisation, therefore reducing the overall cost associated with MUS [17]. Based on those experiences, this study aimed to develop recommendations for commissioners with respect to an evidence-based and clinically desired integrated service model for patients with FSS.

## Methods

A multidisciplinary expert group was established in Bedfordshire in 2020 by the Medical Director for Research & Innovation from ELFT, inviting representatives from all healthcare provider organisations with relevance for the treatment of patients with functional somatic syndromes in the community (see authors list, in addition staff from the Bedfordshire Chronic Fatigue Syndrome Service and the Improving Access to Psychological Therapy Service).

This expert group aimed to map existing services, to review examples of services where elements of care are provided in a holistic, integrative fashion and to centrally consider the views of people with lived experience of functional somatic symptoms.

### Service mapping

The clinical expert group conducted a mapping exercise of all services that provide elements of healthcare for this particular patient cohort. This was carried out to understand the existing service elements and problems in care delivery, to discover underlying interrelationships and structures as a basis for a shared model of the clinically desired system, and to understand how the system structure creates observable outcomes. Team members from the respective providers were also asked to identify their vision for an updated service model. The expert group defined the main questions and started with the construction of a visual map of the current complex system of care; in the second step, the group reflected on this service map, described current referral/treatment pathways, and identified strengths and weaknesses with those services operating separately. The group considered this in the context of the available evidence base and the recommendations obtained from the service user lived experience focus group. Third, the group strived to achieve consensus and to develop a vision for the future.

### Focus group with service users

To better understand the experience of the healthcare journey by patients who had been managing functional somatic symptoms and to develop lived experience recommendations to improve the service pathway within Bedfordshire, we conducted a focus group with patients. Recruitment to the focus group used convenience sampling, through staff within the expert group who work with this patient population. They were asked to identify potential participants for the focus group. In addition, the lead GP from Leighton Road Surgery in Bedfordshire was asked to identify potential participants from the surgery caseload. Willing participants were then contacted via telephone by the ELFT People Participation Lead for Bedfordshire Community Health Services (CG), who provided information about the purpose of the focus group, the logistics of the day, the need for confidentiality, and reward and recognition for taking part. This was also an opportunity for all potential participants to ask any questions and feel prepared before deciding to take part in the focus group.

The semi-structured online focus group format followed a topic guide specifically developed for this study (see supplementary file); the topic guide was not pilot tested. Based upon discussions within the expert group, we hypothesised that the following themes may arise: belief, information, length of journey, diagnosis, pathway (care and treatment/processes), and communication (including language and terminology). These hypotheses formed the basis of the questions chosen for the interview topic guide, shown below.

To better understand the existing pathway, we asked:

1. What has been good about your experience of services/care and treatment to date?
2. What has not been good about your experience of services/care and treatment to date?
3. Where or who did you first go to for help when your symptoms started?
4. What happened when you first went to your GP?

To better understand the impact of people’s experiences, we asked:

5. What impact has good experiences had on you?
6. What impact has bad experiences had on you?

To guide improvements and shape the future pathway, we asked:

7. What would you have liked to have happened?
8. What should the pathway look like?

CG contacted the participants prior to the focus group and explained her involvement and personal interest in the study as an expert by experience. All participants provided informed consent; the focus group was delivered online on a singular occasion and lasted for two hours (including a 15-minute break). It was facilitated by author CG with a staff member supporting any technical issues and offering support around navigating the chosen virtual platform.

The discussion of the focus group was audio-recorded and transcribed for thematic analysis by author CG (female); transcripts were not returned to participants for comments. Ethical approval for this service evaluation was obtained from the East London NHS Trust Governance and Ethics Committee for Studies and Evaluations (GECSE).

A realist epistemology, grounded in the pragmatism of service improvement within the NHS was adopted as the theoretical stance during analysis. Thematic analysis [20] was conducted on a question-by-question basis (by CG), this allowed for an inductive approach (per question) which was set within a wider theoretical or deductive frame (i.e. the question format of the whole focus group). This method ensured that the identified themes remained strongly linked to the data and that the lived experience of the focus group members was privileged. Furthermore, interpretation was minimised and themes remained at a semantic level. Analysis began with data familiarisation through reading and re-reading the transcript, line by line coding and initial theme development followed, and then, reviewing the themes for completeness through connecting back to the primary data in the transcript. For each question and answer pair, a thematic map was developed, which showed the hierarchy of themes and relational factors between themes. The prevalence of identified codes was noted, but not used to weight the themes. Using the thematic maps enabled clear identification of themes additional to those hypothesised by the expert group; though these were not integrated in the overall analysis. A further stage of synthesis of all themes from each thematic map resulted in meta-themes which represent this group’s core lived experiences.

## Results

*Service mapping (the Central Bedfordshire population 294,200 according to the 2021 census) to identify care options available for patients with functional somatic symptoms*:

Existing treatment for people with medically unexplained persistent symptoms/ Bodily Distress Disorder involved six different (at times overlapping) services across the county, as follows:

*Primary care services* consisted of 11 primary care networks with 43 surgeries. GP surgeries were often the first point of access for patients with somatic symptoms, although surgeries varied in their approach to dealing with patients who presented with FSS (or diagnosis of MUS/PPS), and no surgery provided a dedicated service for these patients.

*Luton & Bedford Liaison Psychiatry Services* provided assessments, treatment recommendations and general management plans for inpatients at Luton & Dunstable and Bedford hospitals who presented with a combination of mental and physical health problems. The service provides signposting and connecting with other services, and optional brief psychosocial interventions are offered. Although the Service was not specifically commissioned to provide for patients with FSS, psychiatrists contributed to multidisciplinary team meetings at Bedford Hospital where very complex cases with chronic pain, eating disorder issues, substance misuse issues, recurrent admissions, etc., were discussed.

The *Pain Psychology/Pain Management Team* at Circle Integrated Care (Bedfordshire), in partnership with the Essex Partnership University NHS Foundation Trust (EPUT), provided patients with musculoskeletal (MSK) conditions with outreach to various settings, including outpatient clinics, GP surgeries, and community venues. It also included a pain management service for patients where other physical/medical interventions have been tried or were not considered appropriate and where the pain has been manifesting for three to six months or longer. A clear emphasis was placed on ‘management’ and not on ‘cure’. Additionally, the Pain Psychology Service offered a one-to-one assessment, psychoeducation and brief psychological therapy (on average, six sessions with a range from one to twelve sessions) for some patients based on cognitive behaviour therapy (CBT). There was also a group-based pain management programme (PMP, based on CBT) with six sessions of PMP (two to three hours per session) and one three-month follow-up session. This was led by the psychologist and specialist pain physiotherapist, with the pain nurse also involved in one or two sessions.

*Bedfordshire Chronic Fatigue Syndrome (CFS) Service* The CFS service of Bedfordshire Community Health Services provided specialist multidisciplinary team (MDT) care for patients with at least four months of chronic and significant fatigue, where the fatigue was persistent or relapsing and was present for at least 50% of the time (as per 2016 guidance). Patients presented with associated symptoms such as joint/muscle pain, reduced memory and concentration, and fatigue substantially impacted daily activities. The small MDT of psychologists, physiotherapists, and occupational therapists provided MDT assessment, collaborative diagnosis and treatment, self-management education, graded exercise therapy, CBT and occupational therapy inputs (service treatment has since changed to ‘Energy Management Principles’).

*Improving Access to Psychological Therapy Service* (IAPT; now: Talking Therapy Service)

Bedfordshire Talking Therapies provide mainly CBT and a range of different therapy modalities through individual or group sessions. The service aims to help people aged 17 and over who are experiencing common mental health problems such as depression and anxiety disorders. In addition, the service also offers help to foster living well with long-term physical health conditions, recognising that mental health can play a role in physical health in relation to the symptoms experienced and the impact this has on people’s lives. Talking therapy services are now also offering treatment to patients with MUS according to the expansion of the remit of the previous “IAPT” service.

*Secondary Care Mental Health Services*: There was no secondary care mental health service in Bedfordshire dedicated to offering treatments for individuals with functional disorders. Only patients with a significant degree of psychiatric comorbidity are accepted by community mental health teams with a focus on providing care for mental health problems. Patients were predominantly seen by liaison psychiatry teams, if referred. Some patients with more severe symptoms and corresponding disability were referred to one of the tertiary neuropsychiatry centres in the country.

Following the visual mapping exercise and sorting of identified themes, a co-created outline for an integrated Functional Somatic Symptom Disorder Care Pathway, which we termed ‘Staff Vision’, was developed as follows:

*Time*: Significantly reduced waiting times lead to patients receiving the right service in a timely manner. Patient reports of being ‘bounced’ between several services and not receiving any targeted intervention to address their needs were reduced. This is also related to early intervention, which has the potential to prevent patients from becoming chronic.

*Communication*: The provision of seamless referral pathways between different service providers, built on improved communication within teams and across services, particularly between primary and secondary care, as well as with community psychology services. At the beginning of the pathway, good-quality MDT assessment with individual collaborative formulations was ensured. During treatment, regular MDT meetings were provided across services to present and discuss cases.

*Barrier-free Care*: For patients to experience seamless integrated service without referral processes between service providers. Ideally, there is no waiting time for patients, which could be addressed using signposting to where patients can receive care more rapidly if one service has no capacity. Accessible and straightforward referral routes and clarity in terms of what each service is offering so that there is no overlap. The implementation of more joint up-working between services, particularly where gaps have been identified in service provision and patients do not meet the criteria for CMHT, Bedfordshire Wellbeing Service/IAPT or specialist pain management/chronic fatigue services.

*Choice*: A range of therapeutic interventions are offered through a range of professional modalities. This includes the introduction of more virtual therapy groups, which have been successful since the COVID-19 pandemic. In addition, a mixture of group and individual therapy provisions was provided, with adequate group therapy space. The expansion of the workforce to routinely include physiotherapists and occupational therapists. Formalising pathways for liaison with specialties such as gastroenterologists and neurologists.

*Continued Professional Development, Evaluation & Research*: Initially, further education of primary care practitioners to identify suitable patients, support positive engagement and refer appropriately. Then, a system of training and development on this subject across all disciplines ensures that best practices are shared with all parts of the healthcare system. The provision of professional forums to discuss complex cases that use all levels of healthcare. Ongoing evaluation of services and patient experiences has the potential for large-scale research to demonstrate impact.

*Clinical Governance*: Within this barrier-free model of care, which spans traditional healthcare boundaries, there are clear operational policies for services, meaning that staff members have a clear remit and that patient expectations can be effectively managed.

### Focus group of people with lived experience

All five participants who expressed an interest were included: four participants were female, and one was male. One participant described their ethnicity as mixed and four stated that they were white. The participants’ age range was from 21 to 60 years old. There was a range in employment status and level of educational attainment amongst the participants.

For brevity within this paper, the full findings will not be reported. An overview of the four meta-themes identified by people with lived experience of a functional somatic symptom condition are shown below:

- Patient perceptions of being both believed and understood by healthcare professionals supported a more positive experience of care. The focus group linked this to effective communication on the part of the professional and highlighted the skill of listening.
- The professional and personal attributes of healthcare professionals, as perceived by patients, also affected the healthcare journey. The GPs, as the first point of contact for patients, played a pivotal role in determining whether these patients had a positive experience and healthcare journey. The focus group linked more positive experiences of care to greater knowledge and understanding of FSS and of potential treatment pathways by healthcare professionals. Furthermore, the group highlighted that their perception of the professional’s attitudes toward FSS directly affected their wellbeing.
- The length of the journey from initial assessment to diagnosis affected health outcomes and experience for these patients, with longer healthcare journeys linked to more negative experiences by focus group members.
- Understanding and accepting their condition was an important aspect of these patients’ healthcare journeys. The focus group considered that being supported by healthcare professionals, both to understand and to accept FSS, was an important part of their experience.

### Recommendations

Following a review of the findings from both the service mapping and from the focus group the following recommendations were suggested. Furthermore, the expert group suggested setting up a county-wide steering group with representatives from all healthcare organisations to provide a context for continuing engagement with commissioners and people with lived experience.

1. *Transparent communication, accountability and coproduction*: Engagement with service users should be sought and achieved through Focus Groups, Surveys, Personal interviews, Open Forum events, Patient Representation on the Steering Group, and direct participation in service-level Quality Improvement projects. The steering group should regularly provide feedback to people’s participation groups about how their contributions have helped shape the project towards an integrated care offer.
2. *The importance of primary care services and empathic/trustful communication for the patient journey and experience*: Reports from patients and professionals indicate that the success of the interactions between these patients and their GPs early on and during the healthcare journey plays a pivotal role in determining how patients judge their experience of care and treatment within this pathway and may impact the length of their health journey and ultimately their health and wellbeing. A training package for all primary care health care workers should address the importance of developing trustful relationships and a shared explanatory model that no longer carries simplified and dichotomic assumptions about the nature of the physical complaints (as being psychological in nature). Instead, the training must provide an up-to-date account of bodily distress disorder symptoms in the context of a contemporary biopsychosocial model. A new service model should consider the positive impact that individual health care professionals can have on the experience of patients on this pathway. Participants in the focus group cited particular healthcare professionals and their ability to listen and ‘believe’ the patient and their support for the patient to understand and accept a diagnosis as key moments in their journey. People with lived experience refer to a predominant model among professionals, suggesting “it’s all in the mind”. Taking a holistic approach and knowledge and understanding the nature of FSS seemed to be the defining characteristics of the HCPs that were mentioned positively. The Focus Group discussions suggested that communication (language and terminology) between healthcare professionals and patients may be pivotal to this population feeling believed and understood. It may also play a role in how patients feel about their diagnosis. Team-based training should emphasise the importance of empathic acceptance and the relevance of the initial consultation, the chosen language to label somatic complaints. The training package must encompass practical steps to foster therapeutic relationships at all levels, including an awareness of key “Do’s and Don’ts according to the literature (Table 1):

**Table 1:** Professional behaviour recommendations for Health Care Professionals providing care for patients with FSS/MUS/PPS/BDD (modified based upon a literature synthesis, e.g. [21–23]

Recommended (Do’s)	Discouraged (Dont’s)
• Acknowledge the symptoms and their severity. Understand the patient and the effect the symptoms are having on them.	• Tell the patient that you can find nothing wrong. (There is something wrong).
• Show patient you believe they have the symptoms. They are real and they are experienced in the body.	• Tell the patient the symptoms are normal. They are not normal for the patient.
• Be honest when a patient has unusual or inconsistent symptoms.	• Reassure repeatedly (never ending cycle).
• Think about how you can empower the patient.	• Tell the patient there is nothing you can do to help.
• Explain the links between physical and psychological stresses with clear and positive language.	• Give results of normal tests and reassure and think that this will help.
• Negotiate a culturally responsive explanation.	• Suggest to the patient that the “real” cause of the symptoms is a psychological problem.
• Normalise: all symptoms are biopsychosocial	•Provide simplified and dichotomic purely psychological or purely somatic explanations for physical symptoms.

3. *Updating knowledge through continuous professional development (CPD) and Curricula amendments*: The focus group findings suggested that knowledge and understanding from healthcare professionals (particularly GPs) may be an area that could significantly impact the healthcare journey of this patient group. GPs should be adequately trained to make a provisional diagnosis of bodily distress disorder according to new criteria in the ICD-11 or the equivalent “somatic symptom disorder” in the DSM-V. Healthcare professionals should be able to understand contemporary biopsychosocial care models and the overlap of functional symptom disorders with conditions such as complex posttraumatic stress disorder.
4. *Systemic issues/the provider collaborative*: Commissioners should consider the inadequate current length of time from first presenting with symptoms to the point of diagnosis; it was strongly emphasised that this gap needs to be reduced for patients on this pathway. The focus group discussions suggested that the length of time to diagnosis may have a negative impact on both patients’ experience and their health and wellbeing (course and prognosis). Service-level interventions must be provided in a seamless, coordinated manner, allowing real-time monitoring and timely communication of all care elements provided. Patients should be given a main point of contact (ideally their primary care practitioner) and communicated clearly to the patients as a comprehensive care plan (akin to the principle of long-term condition management). Integrated care and continuity are key components for patients to feel adequately supported, and service users stressed the fact that repetitive accounts of their symptoms induce a sense of chaos and a feeling of being misunderstood. Information sharing between professionals is crucially important to reduce the need for patients to retell their stories to multiple HCPs. This is of particular importance within primary care if patients are not able to see the same GP.
5. *Treatment and support options*: Developing a shared understanding, mutually acceptable and valid conceptualisation of patients’ somatic complaints is the first and foremost objective in the treatment of FSS conditions. This requires a timely and skilled provision of psychoeducation, utilising established new explanatory frameworks that enable patients to explore the inseparable and situative (embedded) nature of all experiences across the spectrum of mental, somatic and interpersonal phenomena. Reference should be given to the importance and process of patients’ understanding of their condition and the potential this may have in improving both experience and health outcomes. Some patients went on to say that understanding their condition improved their ability to self-manage.

Based upon a clearly communicated working diagnosis, treatment should be offered according to the principles of informed choices, acknowledging that patients have different preferences and characteristics that impact the effectiveness of treatment options. According to the findings of this study, treatment options included in a portfolio for an integrated service, delivered by a multidisciplinary group of health care professionals and for patient’s choice: self-help (sensible literature web sites, handouts); problem solving for social/interpersonal problems; psychoeducation; reattribution approaches, psychological treatment: CBT, psychodynamic psychotherapy, mindfulness/mindfulness-based stress reduction, body-oriented psychological therapy, exercising; progressive muscle relaxation and related techniques; Yoga/Pilates courses; and drug treatments: herbal remedies and antidepressants.

## Discussion

This project enabled a stock-taking analysis of services provided for patients with FSS disorders in the county of Bedfordshire, bringing together people with lived experience (patients) and experts from various services currently involved in the provision of care elements for this patient population: the MSK service, A&E Psychiatric liaison services, Primary Care, Community Health Services, Secondary Care Psychology, Liaison Psychiatry, and IAPT. The network aimed to develop a system proposal for service reconfiguration to create a seamless service with multiple access points and flexible care delivery. The recommendations developed by the FSS care provider network in Bedfordshire are broadly consistent with the recently published national guidance by the National Neurosciences Advisory Group in February 2023 [24] for integrated care of patients with functional neurological disorder (FND). The guidance highlights the importance of peer-support, psychoeducation, multidisciplinary planning of personalised care and integration of health services through pooling of resources from different providers.

Some important variations are required for the provision of an integrated care pathway for FSS patients. Most patients present at the primary care level initially, and the GP is often referred to as the most trusted clinician for these patients, as highlighted by focus group members. The current primary context is, however, significantly compromised in its capacity to provide comprehensive care for long-term conditions. Most GPs receive no specific training in managing medically unexplained symptoms [25] and may lack confidence in exploring the complex holistic nature of functional somatic symptom disorders and the relevant psychological issues potentially involved. The needs of people with FSS vary greatly and often require multidisciplinary input from various therapists. The primary care organisational framework (mostly ten-minute consultation slots) and the large increase in demand in recent years have posed significant challenges. Capitalising on the effective therapeutic relationship patients have established with their GPs, it seems nevertheless advisable if not necessary to provide FSS services at the primary care level. One way of achieving that is the creation of specific clinics with initial assessments and therapeutic elements provided at primary care premises. In many parts of the United Kingdom, newly configured primary care networks and neighbourhood teams can be utilised to identify practices with large enough rooms for group therapy. The recommendations established through the coproduced work of clinical experts and experts by experience suggest a clinical model that operates a single point of access care provided by a team of clinicians working according to a biopsychosocial model and a paradigm of embodied engagement. Integration of care elements involves crucially also transdisciplinary co-creative cooperation and integrated approaches such as psychologically informed physiotherapy or movement therapy.

More specifically, the FSS disorder care pathway can be provided at the primary care level based upon pooled and integrated resources or as specific FSS clinics; either way, the specific health care agenda of patients with a range of functional somatic symptoms makes it necessary for an experienced doctor to provide clinical leadership to provide assurance to both patients and wider systems about concerns that “something serious of medical nature may be missed” (e.g., GPs, pain/MSK specialists or psychiatrists with specific expertise in psychological medicine/liaison psychiatry). The multidisciplinary team needs to integrate the expertise of allied health professionals (physiotherapists, arts therapists and occupational/speech and language therapists) while continuing to operate with a degree of diagnostic uncertainty. The patient’s predominant explanatory belief (“it’s caused by a physical illness not yet detected”) inevitably results in repeated negotiations regarding the necessity for further medical investigations. The input from a senior doctor in the MDT is therefore crucially important in representing the evidence base and medical facts: it has been demonstrated that family physicians demonstrate a high level of accuracy in subjectively recognising functional somatic symptom disorders without the aid of standardised assessments [26]. A systematic review and meta-analysis conducted by Eikelboom et al. [27] identified 16 follow-up studies (1980–2014), and the rate of revised diagnoses for functional somatic symptoms (pain, fatigue, IBS bowel, multiple bodily symptoms) was very low at 0.5%. However, this evidence is of course based on rational and systematic diagnostic testing before a diagnosis of functional disorder is made. The advantage of a primary care-based/aligned service model is therefore the reliable assurance provided to FSS sufferers that medical oversight remains with their GPs/doctors who will respond to any significant change in their presentation, assuring continuity of care and clinical oversight. Anecdotal clinical evidence suggests that doctors tend to avoid assigning diagnostic codes related to the range of functional disorders, which makes it difficult to determine the real scale of the problem in research. Holding care responsibilities for these patients at the team level with distributed and defined roles may make it easier to record FSS conditions in the future. Another important success factor for an FSS service is the continuity of care aspect. Patients with somatic complaints tend to present at various service-level access points at times of subjective crises. An integrated care model should ensure the coordination of care between various provider points, providing seamless and timely information sharing with an identified point of care within the service model so that patients can develop trustful and reliable therapeutic relationships.

**Fig. 1:**
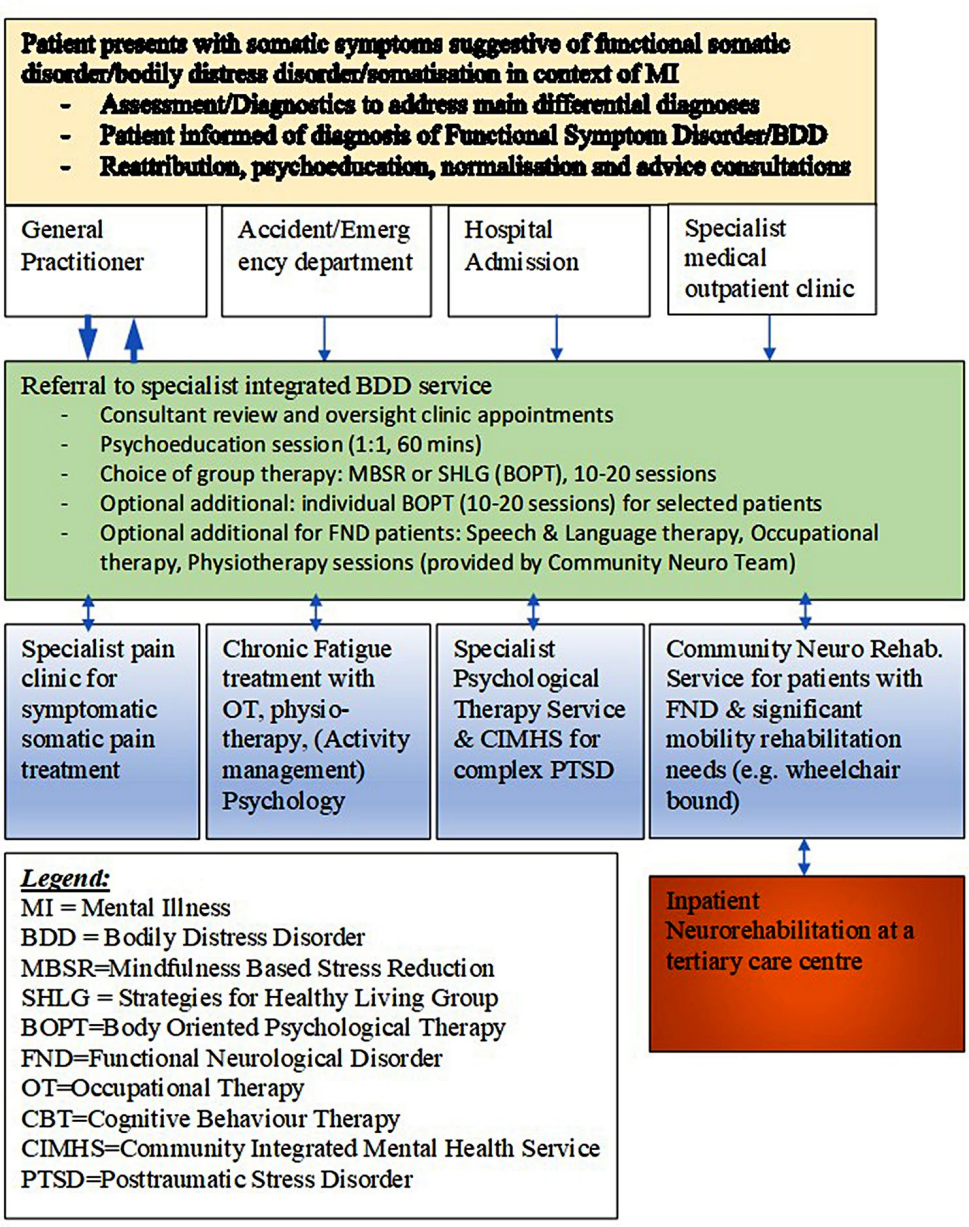
Care pathway illustration

Figure 1 summarises an integrated, desired care pathway model.

Considering the results of a systematic review and metaanalysis for the psychological treatment of somatoform disorders and medically unexplained physical symptoms, it appears increasingly questionable as to whether a standard talking therapy approach is as effective as claimed. For CBT, the standard and most widely applied modality of psychological therapy, moderate improvements in symptom reduction have been reported [28]. Summarising the evidence base for IAPT talking therapies, Geraghty & Scott conclude that “this is not a strong evidence base for IAPT to treat MUS” [14, p. 3]. Recovery rates recorded at IAPT services and the degree of engagement following initial referral both indicate that patient acceptance of this treatment modality remains poor and has suboptimal effects [14]. Based upon developments in cognitive sciences, the embodied nature of thought processes has increasingly been emphasised [29]. Focusing on the most prevalent persistent physical symptom of pain, an updated version of the biopsychosocial model [6, 30] and associated recommendations for holistic treatments therefore refer to new understandings of somatization. Furthermore, according to an empirical study [31], body image is an important feature in patients with somatoform disorders. New treatments have been proposed accordingly, with interventions that operate at the level of the lived body (body/sensory awareness, body cathexis/affect regulation, movement behaviour [29, 30]. One major advantage of these approaches is a better match with the patient perspective and a shared language; current descriptors of complaints presuppose body and mind dualism, whereas the notion of the extended mind [32] asserts that mind is neither body nor brain but rather a complex interplay of those with the environment. A holistic healthcare offer must address binary/Cartesian misconceptions of the nature of functional disorders at the heart of the therapeutic offer. An explanatory model can be communicated to patients in such a way that it validates their experiences (i.e., problems occurring on physical levels in the context of distress). Somatic complaints are not exclusively psychogenic or somatogenic but rather ‘psychosomatic’ in nature. The professional formulation operates comprehensively across domains, allowing for an individualised understanding of the constellation of presenting complaints; this will take into account biological vulnerabilities, biographical factors such as childhood adversity or significant life events, and cognitive and illness behaviour models with corresponding reinforcement of acquired beliefs. A service model that applies a radical embodiment paradigm allows us to focus the therapeutic strategy on important key somatic facts, centring on the sensitisation theory [33]: predisposing biological vulnerability, low pain thresholds, hyperarousal reactions, an amplifying somatic style of coping, alexithymia and focused attention towards distressing bodily sensations (hypervigilance) have been described in the literature [5]. Body-oriented psychological therapies explicitly operate on the level of the ‘lived body’, intrinsically relating to the observation that distress can be expressed through nonverbal (somatic) communication (“body language”, postures, gestures, movements”).

An informal scoping literature review conducted in the context of this study revealed a paucity of primary care-embedded and integrated FSS services, mostly restricted to psychiatric consultation models. Edwards et al. [34] concluded that “GPs’ beliefs about the lack of treatment guidance are consistent with the literature: there are few MUS treatment studies based in primary care” (p. 211). Examples identified as providing integrated care at the primary care level through informal reports refer to an emphasis on self-management, psychoeducation [35] and consultation with experts. Increasingly, multidisciplinary rehabilitation approaches emphasise the important contributions of Allied Health Professionals (see above), with an emphasis on capabilities, physical resilience, stress resistance and autonomic regulation [36]. Few studies have reported the outcomes of integrated service models. The “City & Hackney Primary Care Psychotherapy Consultation Service/PCPCS” report, published by the Centre for Mental Health [37], presented findings from a sample of 282 patients with MUS (49%), personality disorder (51%) or chronic mental health problems (52%). The evaluation demonstrated significant clinical improvements and reductions in NHS service use costs in the 2 years after the start of treatment; however, they calculated the average cost per patient as £1.348 (at the level of cost established for routine care [10]. This is likely related to the fact that the service included mainly patients with high morbidity and severe mental illness. Another holistic care intervention study [38] explored the efficacy of involving relaxation response and resiliency training and showed a significant reduction in healthcare utilisation across clinical services. Röhricht & Elanjithara [16] conducted an analysis of a cohort of MUS patients who were treated with body-oriented psychological therapy (BOPT) in a specialist liaison psychiatry outpatient clinic. Service utilisation in the year following BOPT (A&E attendance and referrals to specialist services) was reported to have significantly decreased by nearly 50%. Similarly, a subsequent cohort study evaluating a novel care pathway for patients with MUS indicated that care pathway implementation at the primary care level results in significant reductions in somatic symptom levels and associated improvements in health-related subjective quality of life [17, 18]. The study demonstrated net savings within the wider care system, particularly within acute care, due to a reduction in unnecessary service utilisation; the calculations suggest that each one pound invested in the care pathway can result in two-three pounds savings elsewhere in the system. Based upon healthcare cost estimations for the study period, the cost per patient treatment was estimated at £228. A corresponding systematic review regarding the cost-effectiveness of interventions for MUS [39] emphasised that group interventions might be more cost effective than individual interventions. Overall, these figures are very encouraging and point towards a highly cost- and clinically effective opportunity for service development across geographic footprints in the context of Integrated Care Systems. These systems allow for sector-based commissioning through provider collaboratives, which is crucial for patients with FSS who have traditionally been neglected in the wider system due to disputes about the nature of their conditions (unsuitable dichotomy of mental versus physical health problems).

Subject to added expertise and resources, those service models might extend to other specific diagnostic groups, such as ‘Chronic Fatigue Syndrome’ and ‘Functional Neurological Disorders’, as well as, to some extent, ‘Long-Covid’ conditions, as they pose significant challenges to health care providers given the diagnostic and aetiological uncertainty. The body-oriented nature of the novel primary care pathway can be an important way to lower the threshold for patients’ acceptance of a holistic care offer because it does not imply psychological causality in the same way as the IAPT/talking therapy model. Instead, an embodied therapeutic experience of contextualising symptoms, exploring their situational nature and the way body and mind operate as a holistic organism, can also become an enabler for subsequent psychotherapeutic exploration of more specific aetiological factors.

In summary, the expert group concluded that the proposed integrated primary care FSS disorder pathway can complement the existing care elements and address a significant gap within the current provider systems with the additional benefit of a promising impact on health economies. Whilst this study aimed to explore healthcare support and treatment offers for patients with functional somatic disorders, pathway development should crucially consider also the importance of promoting resilience, self-efficacy and recovery.

There are however methodological *limitations* to consider for conclusions drawn: due to the fact that this was an unfunded study, only a relatively small group of five patients was recruited through the trust’s people participation team; patients reported to have included collateral information from other patients in their responses, but it remains questionable to what extent their views are representative of the entire patient population across the county. Subsequent research in larger patient samples should capture patient views about service provisions prospectively, utilising the findings of this study to determine hypotheses. The qualitative methods could have been strengthened through initial piloting of the questionnaire to assess suitability, using a team to conduct analysis to enable inter-rater reliability, returning to participants after analysis for member checking and a final stage of integration of findings from both the professional and people with lived experience.

More systematic research is required, guided by people with lived experience, with a view to test the recommendations of this study and their corresponding hypotheses regarding anticipated clinical benefits and cost-effectiveness of delivering an integrated care model for patients with functional somatic symptom disorder.

### Electronic supplementary material

Below is the link to the electronic supplementary material.

Supplementary Material 1

Supplementary Material 2

## Data Availability

Available from the corresponding author on reasonable request.

## References

[1] Burton C, Fink P, Henningsen P, Löwe B, Rief W, EURONET-SOMA Group (2020). Functional somatic disorders: discussion paper for a new common classification for research and clinical use. BMC Med.

[2] NHS England. https://www.nhs.uk/conditions/medically-unexplained-symptoms/ last accessed 19/01/24.

[3] American Psychiatric Association, DSM-5 Task Force. Diagnostic and statistical manual of mental disorders: DSM-5™. 5th ed. American Psychiatric Publishing, Inc.; 2013. 10.1176/appi.books.9780890425596.

[4] World Health Organisation. (2022). ICD-11: International classification of diseases (11th revision). https://icd.who.int/ last accessed 19/01/24.

[5] Henningsen P, Gundel H, Kop WJ, Lowe B, Martin A, Rief W (2018). Persistent physical symptoms as perceptual dysregulation: a neuropsychobehavioral model and its clinical implications. Psychosom Med.

[6] Zipfel S, Löwe B, Giel K, Friederich HC, Henningsen P (2023). Implementing the biopsychosocial model in clinical medicine: a tribute to Giovanni Fava. Psychother Psychosom.

[7] Husain M, Chalder T (2021). Medically unexplained symptoms: assessment and management. Clin Med (Lond).

[8] Jadhakhan F, Lindner OC, Blakemore A, Guthrie E. Prevalence of medically unexplained symptoms in adults who are high users of health care services: a systematic review and meta-analysis protocol. BMJ open. 2019;9(7). 10.1136/bmjopen-2018-027922 PMC6609118 31270115

[9] Lee K, Johnson MH, Harris J, Sundram F (2016). The resource utilisation of medically unexplained physical symptoms. SAGE Open Med.

[10] Konnopka A, Schaefert R, Heinrich S (2012). Economics of medically unexplained symptoms: a systematic review of the literature. Psychother Psychosom.

[11] Bermingham SL, Cohen A, Hague J, Parsonage M (2010). The cost of somatisation among the working-age population in England for the year 2008–2009. Ment Health Fam Med.

[12] Naylor C, Das P, Ross S, Honeyman M, Thompson J, Gilburt H (2016). Bringing together physical and mental health. King’s Fund.

[13] Chew-Graham CA, Heyland S, Kingstone T, Shepherd T, Buszewicz M, Burroughs H (2017). Medically unexplained symptoms: continuing challenges for primary care. Br J Gen Pract.

[14] Geraghty K, Scott MJ (2020). Treating medically unexplained symptoms via improving access to psychological therapy (IAPT): major limitations identified. BMC Psychol.

[15] Van der Feltz-Cornelis CM, Elfeddali I, Werneke U, Malt UF, Van den Bergh O, Schaefert R, Kop WJ, Lobo A, Sharpe M, Söllner W, Löwe B (2018). A European research agenda for somatic symptom disorders, bodily distress disorders, and functional disorders: results of an estimate-talk-estimate Delphi expert study. Front Psychiatry.

[16] Röhricht F, Elanjithara T (2014). Management of medically unexplained symptoms: outcomes of a specialist liaison clinic. Psychiatr Bull.

[17] Röhricht F, Zammit I, Papadopoulos N (2017). Novel primary care treatment package for patients with medically unexplained symptoms: a cohort intervention study. Br J Gen Pract Open.

[18] Papadopoulos N, Burrell C, Smith L, Röhricht F (2017). Therapeutic processes and personalised care in body oriented psychological therapy for patients with medically unexplained symptoms (MUS). Eur J Person Centered Healthc.

[19] Röhricht F, Sattel H, Kuhn C, Lahmann C (2019). Group body psychotherapy for the treatment of somatoform disorder-a partly randomised-controlled feasibility pilot study. BMC Psychiatry.

[20] Braun V (2006). Using thematic analysis in psychology. Qualitative Res Psychol.

[21] Rosendal M, Fink P, Bro F, Olesen F (2005). Somatization, heartsink patients, or functional somatic symptoms? Towards a clinical useful classification in primary health care. Scand J Prim Health Care.

[22] Morriss R, Dowrick C, Salmon P, Peters S, Dunn G, Rogers A, Lewis B, Charles-Jones H, Hogg J, Clifford R, Rigby C (2007). Cluster randomised controlled trial of training practices in reattribution for medically unexplained symptoms. Br J Psychiatry.

[23] Guthrie E (2008). Medically unexplained symptoms in primary care. Adv Psychiatr Treat.

[24] National Neurosciences Advisory Group (NNAG), February. 2023; https://www.nnag.org.uk/optimal-clinical-pathway-adults-fnd-functional-neurological-disorder; last accessed 19/01/24.

[25] Chitnis A, Dowrick C, Byng R, Turner PD, Shiers D. Guidance for health professionals on medically unexplained symptoms. Guidance for health professionals on medically unexplained symptoms. 2011.

[26] Rasmussen NH, Agerter DC, Colligan RC, Baird MA, Yunghans CE, Cha SS (2008). Somatisation and alexithymia in patients with high use of medical care and medically unexplained symptoms. Mental Health Family Med.

[27] Eikelboom EM, Tak LM, Roest AM, Rosmalen JG (2016). A systematic review and meta-analysis of the percentage of revised diagnoses in functional somatic symptoms. J Psychosom Res.

[28] van Dessel N, den Boeft M, van der Wouden JC et al. Non pharmacological interventions for somatoform disorders and medically unexplained physical symptoms (MUPS) in adults. Cochrane Database Syst Rev 2014:CD011142. 10.1002/14651858.CD011142.pub2 PMC10984143 25362239

[29] Gallagher S. Embodied and enactive approaches to cognition. Cambridge University Press; 2023. Jul 31.

[30] Henningsen P (2021). Allgemeine Psychosomatische Medizin: Krankheiten Des verkörperten Selbst Im 21. Jahrhundert [General Psychosomatic Medicine. Disorders of the embodied self in the 21st century].

[31] Scheffers M, Kalisvaart H, Van Busschbach JT, Bosscher RJ, Van Duijn MA, van Broeckhuysen-Kloth SA, Schoevers RA, Geenen R (2018). Body image in patients with somatoform disorder. BMC Psychiatry.

[32] Fuchs T (2020). The circularity of the embodied mind. Front Psychol.

[33] van Ravenzwaaij J, Olde Hartman TC, Van Ravesteijn H, Eveleigh R, Van Rijswijk E, Lucassen PL (2010). Explanatory models of medically unexplained symptoms: a qualitative analysis of the literature. Mental Health Family Med.

[34] Edwards TM, Stern A, Clarke DD, Ivbijaro G, Kasney LM (2010). The treatment of patients with medically unexplained symptoms in primary care: a review of the literature. Ment Health Fam Med.

[35] Pedersen HF, Holsting A, Frostholm L, Rask C, Jensen JS, Høeg MD, Schröder A (2019). Understand your illness and your needs: assessment-informed patient education for people with multiple functional somatic syndromes. Patient Educ Counselling.

[36] Vassilopoulos A, Mohammad S, Dure L, Kozlowska K, Fobian AD (2022). Treatment approaches for functional neurological disorders in children. Curr Treat Options Neurol.

[37] Parsonage M, Hard E, Rock B. Managing Patients with Complex Needs: Evaluation of the City and Hackney Primary Care Psychotherapy Consultation Service. 2014. https://repository.tavistockandportman.ac.uk/880/1/Managing_patients_complex_needs.pdf; last accessed 19/01/2024.

[38] Stahl J, Dossett M, Lajoie A (2015). Relaxation response and resiliency training and its effect on healthcare resource utilization. PLoS ONE.

[39] Wortman MS, Lokkerbol J, van der Wouden JC, Visser B, van der Horst HE, Olde Hartman TC (2018). Cost-effectiveness of interventions for medically unexplained symptoms: a systematic review. PLoS ONE.

