# Supplementary material for: Integrated care model for patients with functional somatic symptom disorder – a co-produced stakeholder exploration with recommendations for best practice
Source: BMC Health Serv Res. 2024 Jun 3;24:698. doi: 10.1186/s12913-024-11130-9 (PMC11145802; doi:10.1186/s12913-024-11130-9)
Supplement: Supplementary file 1 — Supplementary Material 1 [file 12913_2024_11130_MOESM1_ESM.pdf]

FOCUS GROUP FUNCTIONAL SOMATIC SYMPTOM (FSS) SERVICES TOPIC GUIDE

Research aims of the focus group and the interview:

Better understand the experience of the healthcare journey of services users managing multiple medically unexplained / functional somatic symptoms within Bedfordshire.

- What is the experience of using FSS services by people with lived experience (length of journey, diagnoses given and explained, care pathway in term of care and treatment received, communication including language and terminology)?
- What do individuals with lived experience regard as being potentially negative or problematic when using FSS services?
- What do individuals with lived experience regard as being positive and beneficial when using FSS services?
- What impacts and consequences positive or negative experiences have on patients' health condition and recovery?
- What do individuals with lived experience of FSS regard as ideal service provisions (what does good like like)?

Questions to explore those research aims for focus group:

1. What have you found to be good in your experience of managing your symptoms, and care and treatment around managing your symptoms?
2. What have you found not to be good in your experience of managing your symptoms, and care and treatment around managing your symptoms?
3. What happened when you first went to a health professional with your symptoms?
4. What would you have wanted your journey to look like up to this point? What do you want a new bespoke pathway for people with your symptoms, to look like?
5. How does it feel to be presented with an umbrella diagnosis such as Medically Unexplained Persistent Symptoms?
6. Where or who did you first go to, for help when your symptoms started?
7. What happened when you first went to your GP? Impact of experience?
8. What would you have liked to have happened? Suggestions for improvements / vision for the future?
9. What should the ideal pathway look like?

THE FOCUS GROUP

(a) Introduction, overview of focus group activity – first 20 minuntes

Instructions: At the beginning we will introduce ourselves. You will each asked to tell your name and then to describe your experiences in relation to some specific questions.

- Welcome, introduction of researcher and project.
- The interviewer/researcher thanks the participants for their availability, introduces him/herself and explains that: "We are interested in your experiences accessing and receiving healthcare for your persistent somatic symptoms and your suggestions as to how services could be improved".
- The focus group is about identifying and discussing views from service users and the impact if any this has had on their wellbeing.
- Other information provided: The name of the participant will only be known by the researchers and not be revealed to anyone. The interview will take appr. Two hours; it will be audio-recorded, transcribed and analysed to identify common "themes" among different participants.
- The participants will be identified by code only on our records and all potentially identifying information will be removed.
- Then Interviewer invites questions on the project from the participant.

(b) Questions and discussions – 40 minutes

Break (10 minutes)

(c) Resume Questions and discussions – 40 minutes

(d) Thank you, summary of what has been discussed and closure – 10 minutes

- To summarise the points emerged from focus group discussion.
- To ask participant in turn if he/she agrees with the summary provided and if there is anything he/she wishes to add.
- To thank participant for his/her contribution.
- To inform participants that if they want to withdraw from the study, their data will not be used.
